# Synthesis, crystal structures and docking studies of 2,7-diphenyl-1,4-diazepan-5-one derivatives

**DOI:** 10.1186/s13065-015-0094-3

**Published:** 2015-04-08

**Authors:** Maheshwaran Velusamy, Sethuvasan Sreenivasan, Ravichandran Kandasamy, Ponnuswamy Subbu, Sugumar Paramasivam, Ponnuswamy Mondikalipudur Nanjappagounder

**Affiliations:** Centre of Advanced Study in Crystallography and Biophysics, University of Madras, Guindy Campus, Chennai, 600 025 India; PG & Research Department of Chemistry, Government Arts College (Autonomous), Coimbatore, 641 018 India; Department of Physics, Kandaswami Kandar’s College, Velur, Namakkal 638 182 India

**Keywords:** 1,4-diazepine(DAP), Peptidomimetics, Hepatitis C virus (HCV), NS5B RNA polymerase

## Abstract

**Background:**

1,4-Diazepine derivatives are the seven membered, nitrogen containing heterocyclic ring systems possessing a wide range of therapeutic applications. 1,4-Diazepines attracted the attention of chemists and druggists due to their biological and medicinal properties, such as antimicrobial, anti-HIV and anticancer activities. Herein, we report the preparation, crystal structure determined by X-ray crystallographic methods and docking of the molecules with the potential target protein NS5B RNA polymerase.

**Results:**

The crystal structures and conformational studies of 1,4-diazepine [*t*-3, *t*-6-dimethyl-*r*-2,*c*-7-diphenyl-1,4-diazepan-5-one(DIAZ1)] and its nitroso derivative [*t*-3, *t*-6-dimethyl-1-nitroso-*r*-2,*c*-7-diphenyl-1,4-diazepan-5-one(DIAZ2)] are reported. The analyses of the molecules reveal that the seven membered diazepine ring systems adopt chair and boat conformations in compounds DIAZ1 & DIAZ2, respectively. In DIAZ2, the oxygen O2A is disordered over two positions with the refined occupancies of 0.792(7): 0.208(7) in the nitroso group. In both DIAZ1 & DIAZ2, the symmetry related molecules form a hetero/homo-dimer through N-H…O hydrogen bonds.

**Conclusion:**

In this study, the crystal structures of two new 1,4-diazepines, namely *t*-3, *t*-6-dimethyl-*r*-2,*c*-7-diphenyl-1,4-diazepan-5-one and *t*-3, *t*-6-dimethyl-1-nitroso-*r*-2,*c*-7-diphenyl-1,4-diazepan-5-one were synthesized and characterized by X-ray crystallographic methods. The docking studies show that the compounds inhibit at the active site of the target protein and can be utilized as potential drug molecules. In both the compounds, N-H…O hydrogen bonds lead to dimer formation. In DIAZ2, additionally a couple of C-H…O interactions are noted between the molecules.

**Graphical Abstract:**
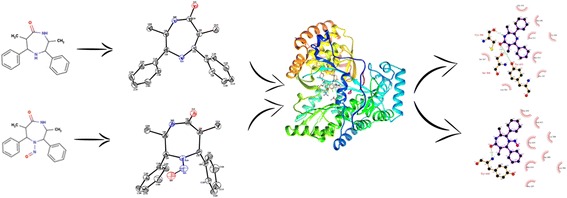
Structure and docking studies of 1,4-diazapine derivatives.

**Electronic supplementary material:**

The online version of this article (doi:10.1186/s13065-015-0094-3) contains supplementary material, which is available to authorized users.

## Background

1,4-Diazepine derivatives are the seven membered, nitrogen containing heterocyclic ring systems possessing a wide range of therapeutic applications. These derivatives are predominantly used in the inhibition of signals in the central nervous system which is useful for the synthesis of psychoactive drugs [1]. 1,4-Diazepines are widely used in the field of peptidomimetics as potential mimetic and molecular scaffolds [2,3]. Analogs of 1,4-diazepine nucleoside with the protected sugar moiety have been made as possible agents against HIV-1 and HIV-2 viruses [4]. 1,4-Diazepines attracted the attention of chemists and druggists for their biological and medicinal activities, such as antimicrobial [5], anti-HIV [6], herbicidal [7], psychotropic [8] and anticancer [9] activities. Also 1,4-diazepines act as antagonists of platelet activation factor (PAF) [10]. 1, 4-Diazepines play prominent roles in the field of medicinal chemistry because it is the core moiety used for the synthesis of various drug molecules like, dibenzepine, clozapine, brotizolam and zometapine. In view of above said importance, 1,4-diazepine derivatives were synthesized and the crystal structures were determined. The docking studies of the above derived molecules were carried out with the targeted protein NS5B RNA polymerase.

### Experimental

Noller and Baliah [11], developed a novel method which involves the condensation of diethyl ketone with aromatic aldehyde along with ammonium acetate in the presence of ethanol medium leading to the formation of the compound *t*-3, *t*-5-dimethyl-*r*-2,*c*-6-diphenylpiperidin-4-one **(1)**.

### Preparation of DIAZ1

To a cooled *t*-3, *t*-5-dimethyl-*r*-2,*c*-6-diphenylpiperidin-4-one **(1)** [1.40 g, 5 mmol] solution in ether (20–30 ml) in an Erlenmeyer flask, was added conc.HCl until precipitation of the white solid was completed (5–10 min). The solid was then collected, washed with ether and dried. Recrystallization of the solid mass from ethanol afforded colorless crystals of **(2)** (Yield: 1.48 g, 93.67%), mp 226–228°C [11].

**Scheme 1:**
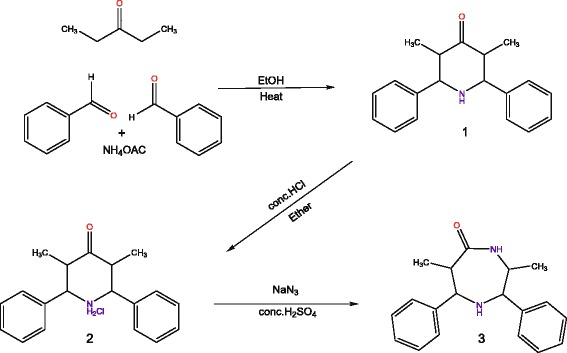
**Reaction schemes showing the synthesis of the compound (DIAZ1).**

Dry**,** powdered *t*-3, *t*-5-dimethyl-*r*-2,*c*-6-diphenylpiperidin-4-one hydrochloride**(2)** [1.58 g, 5 mmol] was added in portions to cold (5°C) conc. H_2_SO_4_ (10–15 ml) in an Erlenmeyer flask equipped with a mechanical stirrer. After the dissolution of **(2)** was complete, the temperature of the solution was allowed to rise 25°C. Then, NaN_3_ (0.65 g, 10 mmol) was added in portions of 0.1 g, with vigorous stirring and the addition was continued for 1 h. Nitrogen gas evolution was observed during each addition. The resulting solution was poured into crushed ice and stirred well with a glass rod and cold sodium hydroxide solution (2 M) was added slowly with stirring until the pH was 8. The white solid separated out was kept in the beaker at room temperature overnight and collected, washed free of sodium hydroxide and dried. The dried solid was dissolved in ethanol and filtered through a fluted filter paper and the solution was concentrated *in vacuo* to 10 ml. The solution was kept aside for crystallization. The crystals of *t*-3, *t*-6-dimethyl-*r*-2,*c*-7-diphenyl-1,4-diazepan-5-one **(3)** were obtained and then recrystallized again from dichloromethane and petroleum ether (60–80°C). (Yield: 1.10 g (74.83%) of colorless solid, mp 178–180°C) [12] [Scheme 1].

### Preparation of DIAZ2

**Scheme 2:**
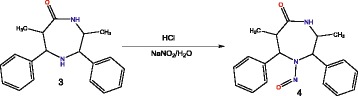
**Reaction schemes showing the synthesis of the compound (DIAZ2).**

Dry, powdered *t*-3, *t*-6-dimethyl-*r*-2,*c*-7-diphenyl-1,4-diazepan-5-one **(3)** [1.47 g, 5 mmol] was dissolved in ether (100 ml), and HCl was passed through the solution until white solid precipitation of hydrochloride of **(3)** was complete. The solid was separated by filtration, washed with ether and dried. The dry, powdered hydrochloride of (**3**) was added, in portions of 0.1 g, to a water-ethanol mixture (water 10 ml; alcohol 10 ml) at 0–10°C in a round-bottom flask and the reaction mixture was equipped with magnetic stirrer. The contents were stirred well until the solid was dissolved. While stirring, a solution of NaNO_2_ (1.38 g, 20 mmol) in water (10 mL) was added dropwise over a period of 1 h at 0–10°C. The precipitated white solid was filtered through a Buchner funnel, washed with water, and the organic extracts were combined, dried over Na_2_SO_4_. The sample *t*-3, *t*-6-dimethyl-1-nitroso-*r*-2,*c*-7-diphenyl-1,4-diazepan-5-one**(4**) recrystallized from dichloromethane and petroleum ether (60–80°C). (Yield: 1.28 g (79.01%) of colorless needles, mp 215–217°C) [12] [Scheme 2].

## Results and discussion

**Figure 1:**
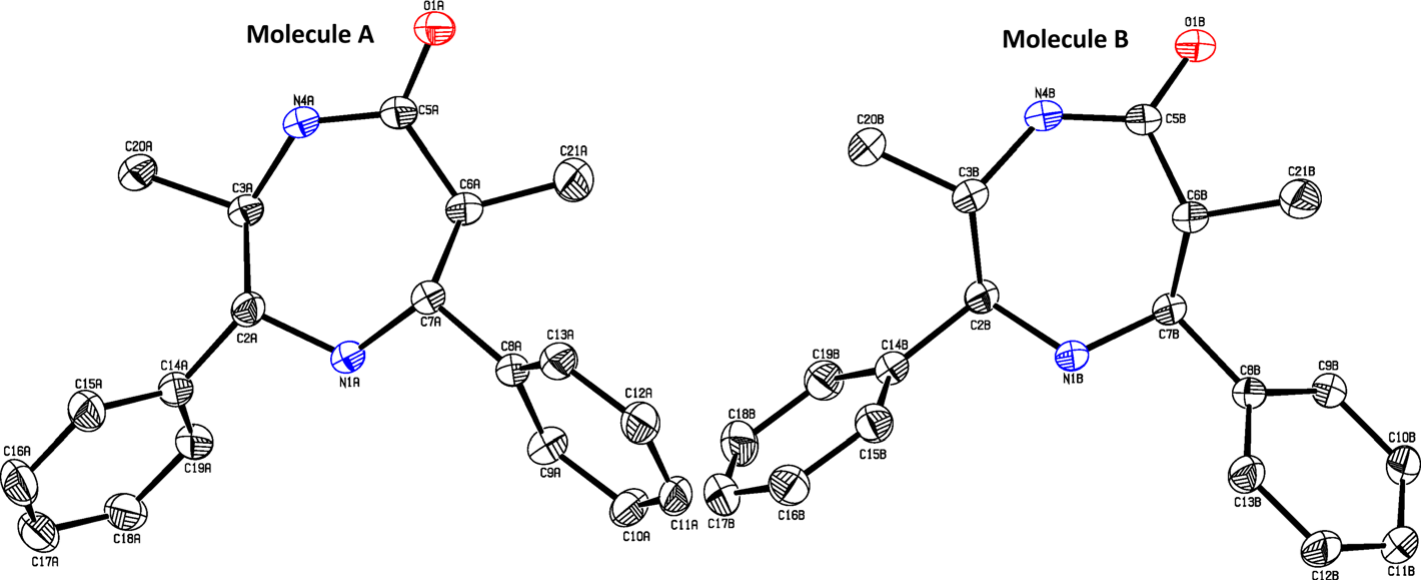
**The molecular structure of DIAZ1 with the atomic numbering and displacement ellipsoids drawn at 20% probability level.**

**Figure 2:**
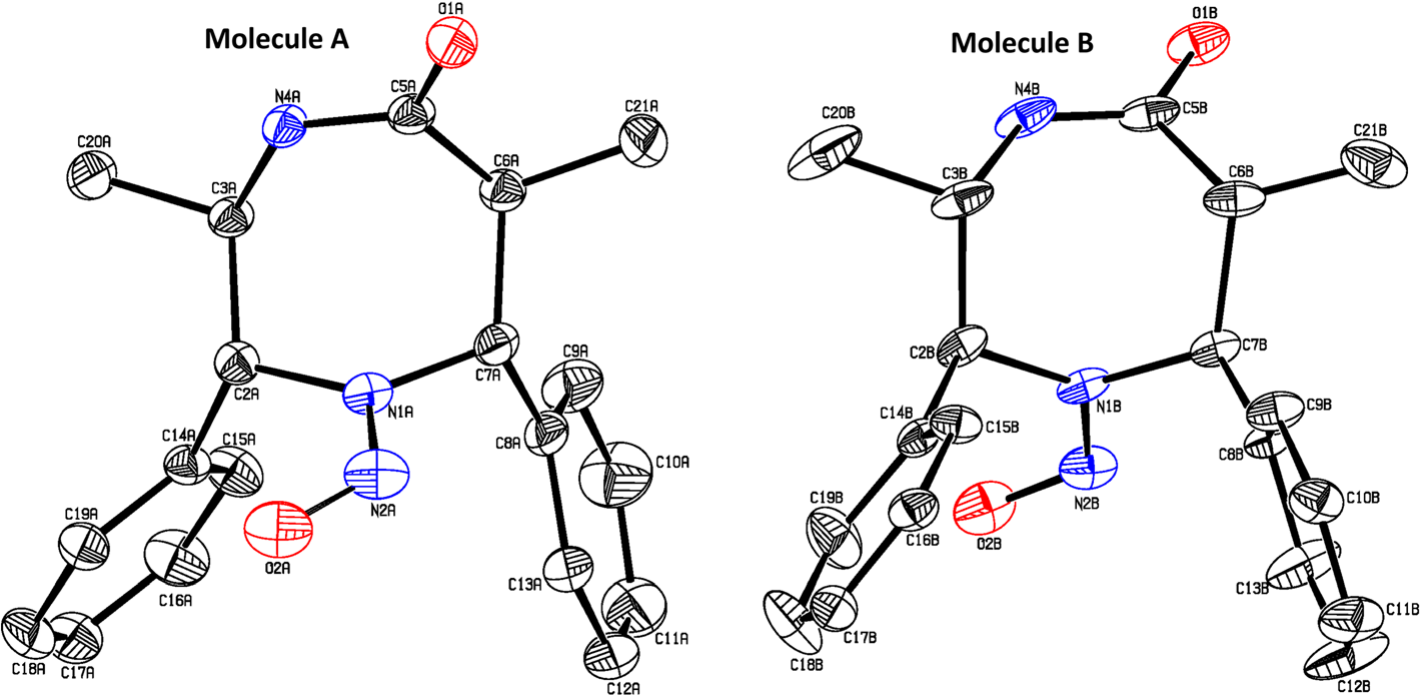
**The molecular structure of DIAZ2 with the atomic numbering and displacement ellipsoids drawn at 20% probability level.** The atom O2A was disordered in the molecule A. The disordered atom O2A’ and all H atoms have been omitted for clarity.

The ORTEP plots [13] for the molecules DIAZ1 and DIAZ2 are shown in Figures 1 and 2. The crystal data, experimental conditions and structure refinement parameters for DIAZ1 and DIAZ2 are presented in Table 1. There are two crystallographically independent molecules in the asymmetric unit in both the compounds DIAZ1 and DIAZ2.

**Table 1:** **Crystal data**

**Parameters**	**DIAZ1**	**DIAZ2**
Empirical formula	C_19_ H_22_ N_2_ O	C_19_ H_21_ N_3_ O_2_
Formula weight	294.39	323.39
Temperature	293(2) K	293(2) K
Wavelength	0.71073 Å	0.71073 Å
Crystal system, space group	Triclinic, Pī	Triclinic, Pī
Unit cell dimensions	a = 11.1980(5)Å α = 93.151(5)°	a = 9.1789(6)Å α = 89.255(4)°
	b = 12.2910(8)Å β = 97.650(3)°	b = 12.7776(9)Å β = 86.758(3)°
	c = 13.3610(4)Å γ = 92.660(4)°	c = 16.0615(10)Å γ = 70.507(4)°
Volume	1817.1(2) Å^3^	1772.9(2) Å^3^
Z, Calculated density	4, 1.076 Mg/m^3^	4, 1.212 Mg/m^3^
Absorption coefficient	0.067 mm^−1^	0.080 mm^−1^
F(000)	632	688
Crystal size	0.25 × 0.26 × 0.30 mm	0.23 × 0.25 × 0.27 mm
Theta range for data collection	1.66 to 28.48°	1.27 to 26.82°
Limiting indices	−15 < =h < =14, −16 < =k < =16,-16 < =l < =17	−11 < =h < =11, −15 < =k < =16, −20 < =l < =19
Reflections collected/unique	30381/8888 [R_int_ = 0.0407]	24353/7277 [R_int_ = 0.0344]
Completeness to theta	96.80%	96.10%
Refinement method	Full-matrix least-squares on F^2^	Full-matrix least-squares on F^2^
Data/restraints/parameters	8888/0/401	7277/3/447
Goodness-of-fit on F^2^	0.957	0.997
Final R indices [I > 2σ (I)]	R1 = 0.0595, wR2 = 0.1687	R1 = 0.0570, wR2 = 0.1479
R indices (all data)	R1 = 0.1369, wR2 = 0.1912	R1 = 0.1163, wR2 = 0.1919
Largest diff. peak and hole	0.377 and −0.367 e.Å^−3^	0.363 and −0.194 e.Å^−3^

(See: Additional files 1 and 2 for CIF of DIAZ1 and DIAZ2).

A study on asymmetry parameters, torsion angles and least-squares planes reveal that the seven membered diazepine rings in DIAZ1 adopt chair conformation whereas the ring in DIAZ2 assume boat conformation (Tables 2 & 3). The altered boat conformation adopted in DIAZ2 is due to the nitroso group substitution at N1A & N1B positions of the molecule(s).

**Table 2:** **Asymmetry parameters and torsion angles for diazepine ring**

		**DIAZ1**		**DIAZ2**	
		**Mol. A**	**Mol. B**	**Mol. A**	**Mol. B**
**Asymmetry parameters**	q2 (Å)	0.387(2)	0.430(2)	1.054(2)	1.017(4)
	q3 (Å)	0.679(2)	0.700(2)	0.141(2)	0.128(3)
	ϕ2 (°)	17.4(3)	−172.7(3)	171.0(1)	176.2(2)
	ϕ3 (°)	176.5(2)	−4.3(2)	−1.9(1)	−8.5(4)
	Q (Å)	0.781(2)	0.821(2)	1.063(2)	1.025(3)
**Torsion angles(°)**	C7-N1-C2-C3	65.2(2)	−66.9(2)	35.5(3)	41.2(4)
	N1-C2-C3-N4	−68.7(2)	73.4(2)	47.9(2)	39.1(4)
	C2-C3-N4-C5	64.9(3)	−64.5(3)	−74.4(3)	−72.1(5)
	C3-N4-C5-C6	−7.6(3)	0.1(3)	−6.8(3)	−2.0(5)
	N4-C5-C6-C7	−59.7(2)	66.5(2)	72.1(3)	70.1(4)
	C5-C6-C7-N1	86.3(2)	−86.3(2)	−30.4(3)	−34.4(4)
	C6-C7-N1-C2	−76.1(2)	74.1(2)	−48.1(3)	−45.8(4)

**Table 3:** **Least-squares plane calculation for DIAZ1 & DIAZ2**

	**Plane**	**m1**	**m2**	**m3**	**D**	**Atom**	**Deviation (Å)**
**DIAZ1**	**Molecule A**	0.301(1)	−0.116(1)	−0.946(0)	−1.683(9)	C2A*	−0.060(2)
						C3A*	0.051(2)
						C6A*	−0.072(2)
						C7A*	0.068(2)
						N1A	−0.668(2)
						N4A	1.017(2)
						C5A	1.001(2)
	**Molecule B**	−0.993(0)	−0.111(1)	−0.040(2)	−0.234(8)	C2B*	−0.038(2)
						C3B*	0.039(2)
						C6B*	−0.047(3)
						C7B*	0.037(2)
						N1B	−0.672(2)
						N4B	1.062(2)
						C5B	1.090(3)
**DIAZ2**	**Molecule A**	−0.231(1)	0.771(1)	−0.593(1)	4.401(1)	C2A*	0.063(2)
						C3A*	−0.063(2)
						C6A*	0.069(2)
						C7A*	−0.067(2)
						N1A	0.391(2)
						N4A	1.077(2)
						C5A	1.240(2)
	**Molecule B**	0.223(1)	−0.781(1)	−0.583(2)	−14.533(2)	C2B*	−0.021(3)
						C3B*	0.025(4)
						C6B*	−0.025(3)
						C7B*	0.018(3)
						N1B	−0.403(2)
						N4B	−1.075(3)
						C5B	−1.173(3)

The equation of the plane: m1x + m2y + m3z - D = 0. Where, m1, m2, m3 and D are constants.

*Atoms are included in the plane calculations.

In DIAZ1 & DIAZ2, the planar phenyl rings attached at C2 & C7 and the keto oxygen at C5 of the diazepine ring occupy equatorial orientation. The sum of the bond angles around the hetero nitrogen atoms N [N1A & N1B and N4A & N4B] in both DIAZ1 & DIAZ2 show that the atoms are in *sp*^*2*^ hybridized state.

**Figure 3:**
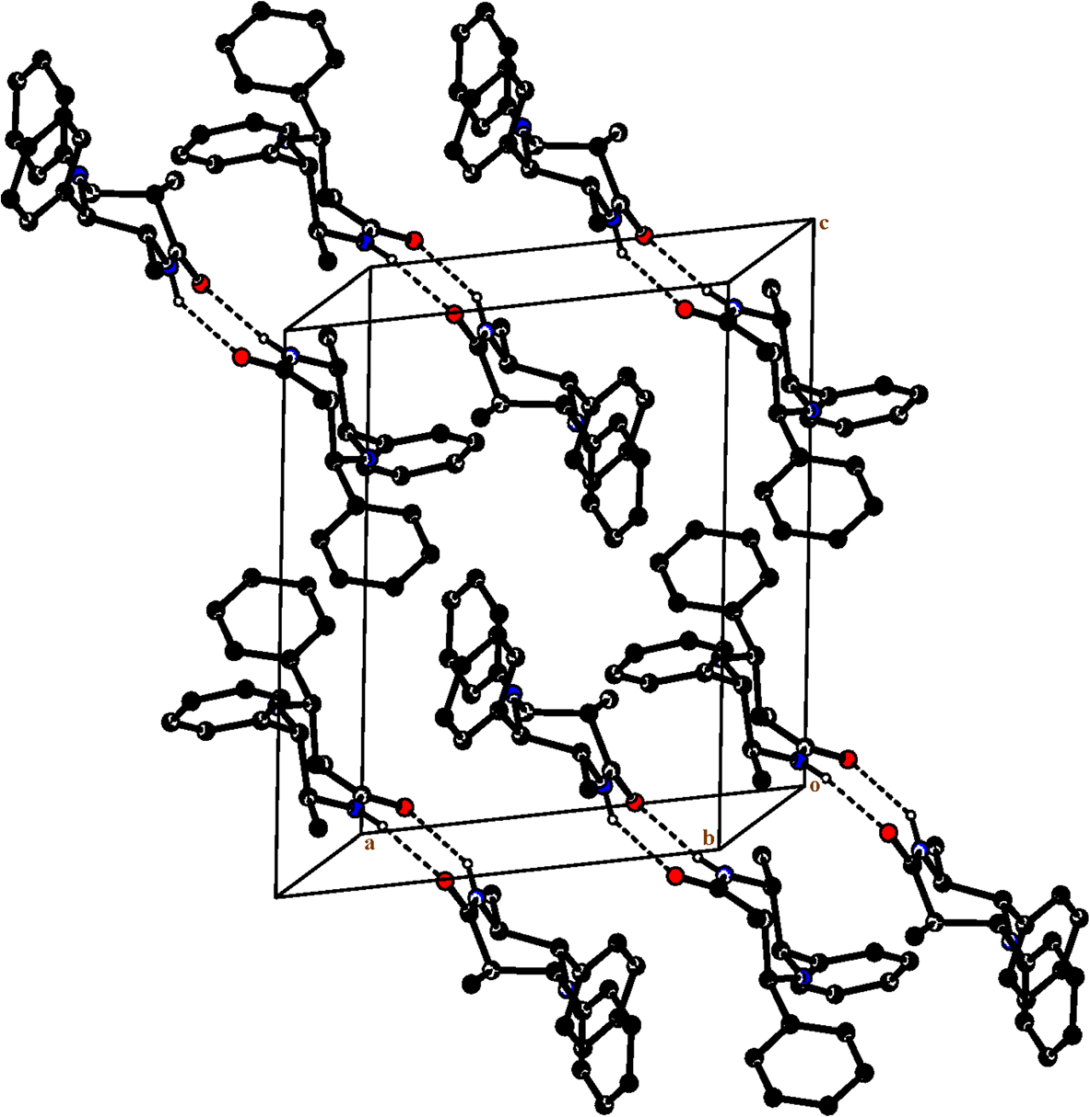
**The packing of the molecules in the unit cell, viewed down the** ***b*** **-axis.** Dashed lines represent hydrogen bonds. H-atoms not involved in hydrogen bonding are omitted for clarity (DIAZ1).

**Figure 4:**
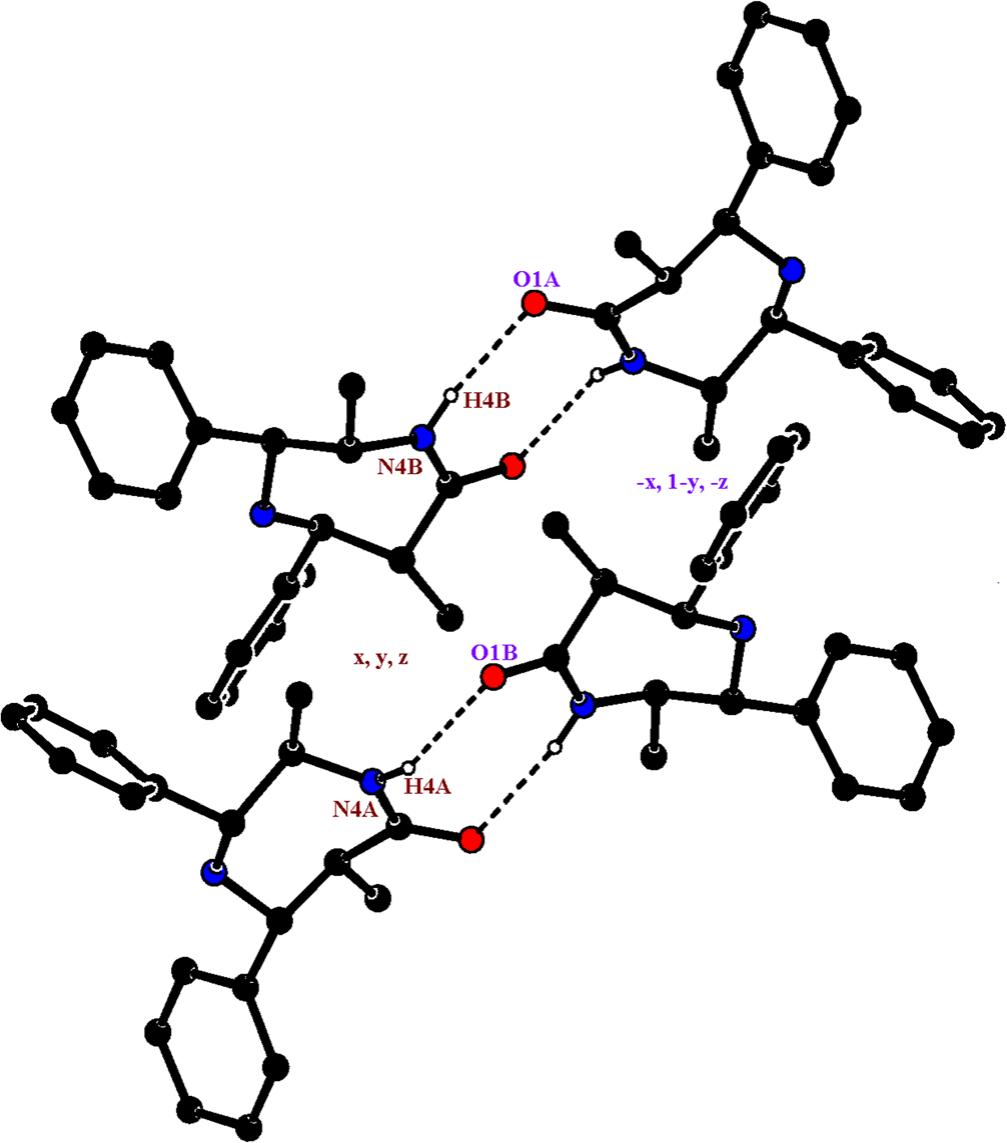
**The hetero-dimer formation of the molecules with the symmetry related ones in DIAZ1.**

In DIAZ2, the atom O2A of nitroso group is disordered over two positions with refined occupancies 0.792(7):0.208(7). The nitroso group is coplanar with the best plane of the diazepine ring as can be seen from the torsion angle values of [C7A-N1A-N2A-O2A & C7A-N1A-N2A-O2A’=] 173.0(2) & 1.7(7)°, respectively. The packing of the molecules in DIAZ1 viewed down *b*-axis is shown in Figure 3. The molecules at (x, y, z) and (−x,1-y,-z) are linked through intermolecular N-H…O hydrogen bonds into a cyclic pseudo-centrosymmetric R^2^_2_(8) hetero-dimer as shown in Figure 4 [14].

**Figure 5:**
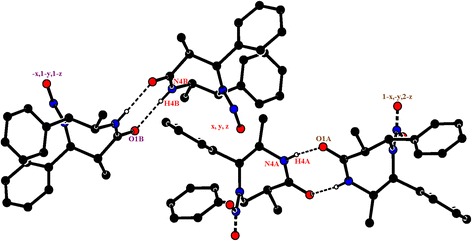
**The homo-dimer formation of the molecules with the symmetry related ones in DIAZ2.**

**Figure 6:**
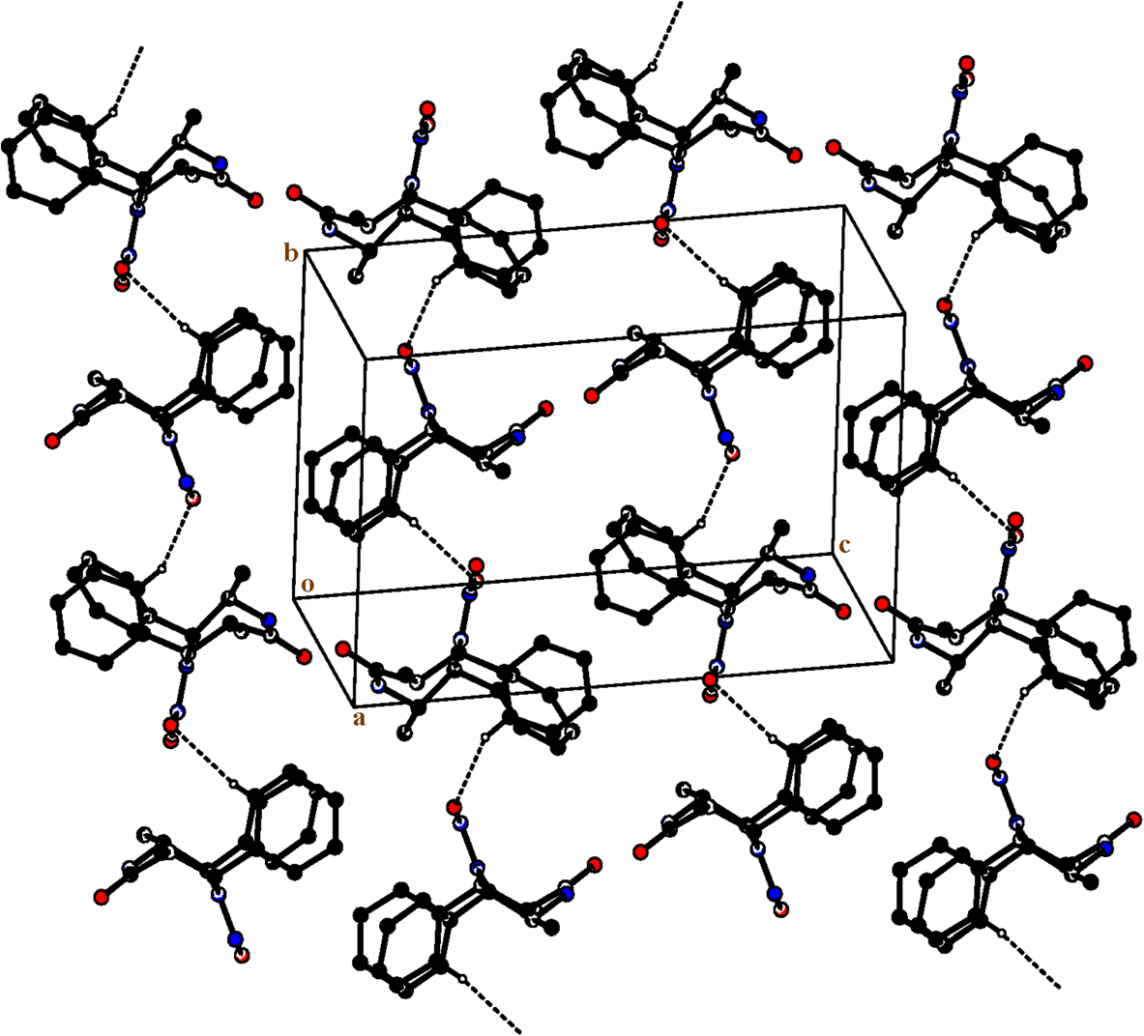
**The packing viewed down the** ***a*** **-axis.** Dashed lines represent hydrogen bonds. H-atoms not involved in hydrogen bonding are omitted for clarity (DIAZ2).

In DIAZ2, the intermolecular N-H…O hydrogen bonds generate the graph set motif of centrosymmetric R^2^_2_(8) homo-dimer as shown in Figure 5 [14]. The molecules at (x, y, z) and (−x,1-y,1-z) are linked by C-H…O hydrogen bonds and form a C(7) chain running along *b*-axis (Figure 6). A number of N-H…O and C-H…O types of intra and intermolecular interactions form a three dimensional network in the crystal lattice. The relevant details of the intermolecular features of DIAZ1 and DIAZ2 are given in Table 4.

**Table 4:** **Hydrogen-bond geometry (Å, °)**

	**D-H…A**	**d(D-H)**	**d(H…A)**	**d(D…A)**	**<(DHA)**
**DIAZ1**	N4B-H4B…O1A^i^	0.86	2.13	2.985(2)	171
	N4A-H4A…O1B^i^	0.86	2.15	2.913(2)	147
**DIAZ2**	N4A-H4A…O1A^ii^	0.86	2.14	2.978(3)	166
	N4B-H4B…O1B^iii^	0.86	2.03	2.890(4)	178
	C9A-H9A…O2B^iii^	0.93	2.49	3.235(5)	138
	C9B-H9B…O2A^iv^	0.93	2.50	3.387(4)	160

Symmetry equivalent positions:

^i^-x,1-y,-z

^ii^1-x,-y,2-z

^iii^-x,1-y,1-z

^iv^1-x,-y,1-z

### X-Ray Crystallography

Single crystals of the compounds DIAZ1 and DIAZ2 were mounted on a Bruker Kappa APEXII area-detector diffractometer equipped with MoKα (0.71073 Å) radiation [15]. X-ray intensity data were collected for both the compounds at room temperature (293 K). The data reduction and the absorption corrections were carried out using SAINT [15] and SADABS [16] programs. The structures were solved by direct methods using the program SHELXS97 [17]. All the non-hydrogen atoms were refined anisotropically by full-matrix least-squares procedure on F^2^ taking all the unique reflections using SHELXL97 [17]. The hydrogen atoms attached with carbon atoms were placed in their calculated positions and included in the isotropic refinement using the riding model with C–H = 0.93 Å (−CH) or 0.97 Å (−CH2) Å or 0.96 Å (−CH3) Å with U_iso_ (H) = 1.2U_eq_ (parent C atom). In DIAZ1, molecules of solvent were severely disordered, but suitable disorder models could not be found. In order to obtain a better quality refinement, the *SQUEEZE* routine in *PLATON* was used to remove the contribution from the disordered solvent [18]. The least-squares planes, geometrical and puckering parameters of both the compounds were calculated using PLATON software package [19-21].

### Molecular docking studies of diazepine derivatives

Hepatitis C virus (HCV) is a positive sense single stranded RNA virus belonging to the flaviviridae family of enveloped viruses. The hepatitis C viral particle consists of a core of genetic material (RNA), surrounded by a protective shell of protein, and further encased in a lipid (fatty) envelope of cellular material. This protein is processed by host and viral proteases into four structural (Core, E1, E2 and p7) and six nonstructural proteins (NS2, NS3, NS4A, NS4B, NS5A and NS5B) [22]. The objective of the study is to demonstrate that 1,4-diazepines (DAP) bind to the NS5B enzyme and to evaluate whether these DAP molecules can be used as potential drugs for hepatitis C disease.

The diazepine derivatives derived in the present study were analyzed for the binding affinity with NS5B polymerase. The co-crystallization of various dibenzodiazepine with NS5B has already been carried out wherein carbonyl O of the inhibitor forms an intermolecular hydrogen bond interacting with residue TYR 448.

### Target protein and ligand structure preparation

The X-ray crystal structure of NS5B complex (PDB ID: 3CSO) was obtained from the RCSB Protein Data Bank (PDB). Prior to optimizing the protein, water molecules were removed from the crystal structure and partial atomic charges were also assigned according to the force field. Minimization of protein was performed until the average root mean square (rms) deviation of the non-hydrogen atoms reached 0.3 Å using OPLS-2005 force field to remove the steric hindrance under Protein Preparation Wizard of Schrödinger Suite 2011 [23]. The above said force field was used in minimizing the energy of the ligands. The pictorial representation is done using the program LIGPLOT [24]. Ligplot generates schematic diagrams of protein-ligand interactions from the 3D coordinates in a PDB file.

**Figure 7:**
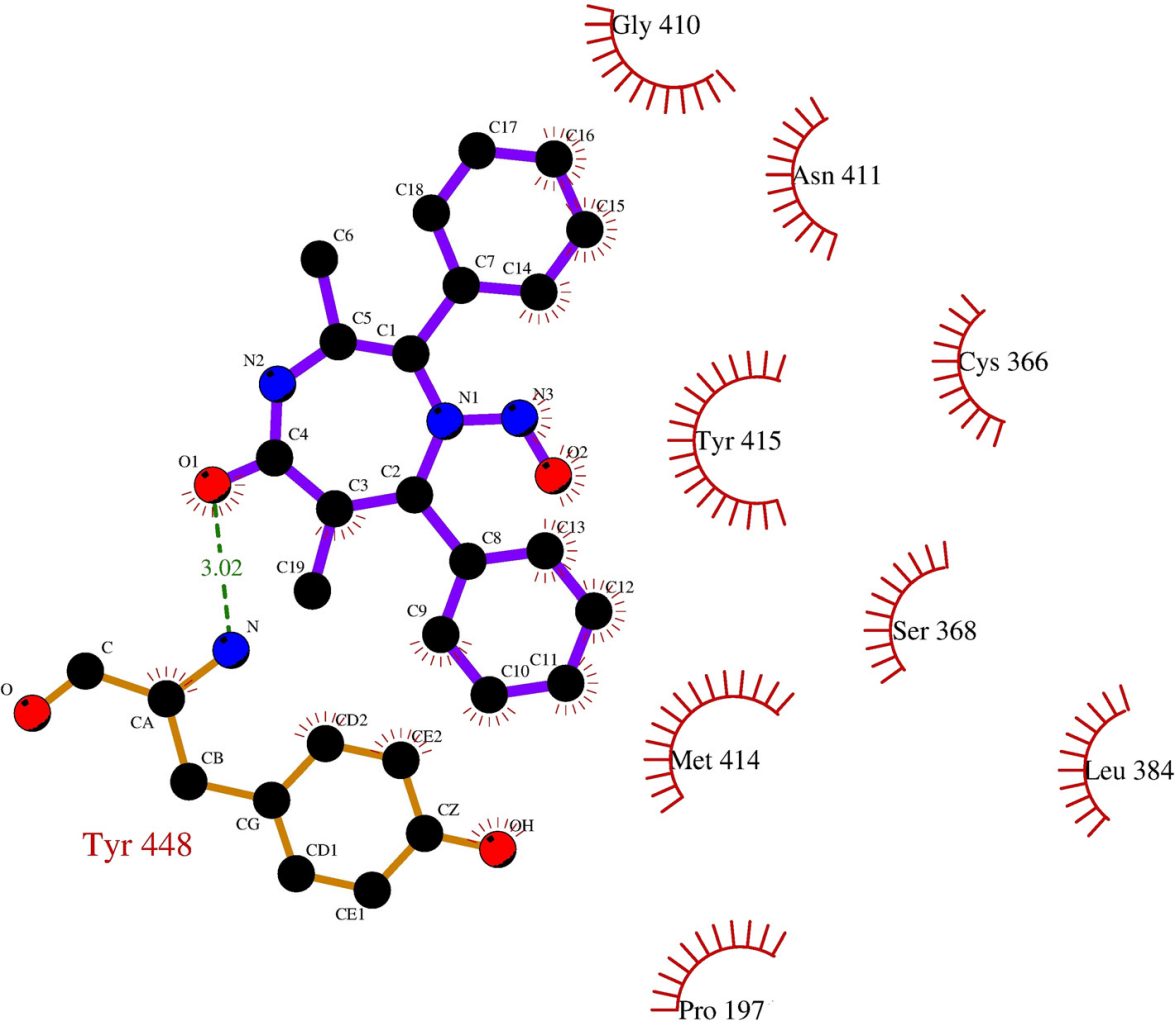
**Ligplot show interactions between DIAZ2 and protein NS5B RNA polymerase.**

The results obtained from this study would be useful in both understanding the inhibitory activity of 1,4-diazepine derivatives and accurately predicting the activities of newly designed inhibitors based on docking scores as well. The two ligands DIAZ1 & DIAZ2 are docked with the NS5B RNA polymerase and compared with the co-crystallized ligand, namely dibenzodiazepine [C_30_H_29_ClN_2_O_3_]. In DIAZ2, the carbonyl O atom interacts with TYR448 as also seen from the dibenzodiazepine (Figure 7). Based on the docking scores and energy values, DIAZ2 has better values when compared with DIAZ1 (Table 5). Although the docking scores for the new inhibitors are slightly inferior, they are achieved with significantly fewer atoms.

**Table 5:** **Score, energy and interactions of DIAZ1 &DIAZ2 with NS5B RNA Polymerase(PDB ID: 3CSO)**

	**Docking score**	**Glide energy (kcal/mol)**	**Type of interaction**	**Bond length (Å)**
**DIAZ 1**	−6.983	−41.286	N-H…O (CYS366)	3.11
			O-H…O (SER368)	2.69
			N-H…O (TYR415)	3.18
**DIAZ 2**	−7.246	−44.008	N-H…O (TYR448)	3.02
**Co-crystal**	−8.645	−60.956	N-H…O (TYR448)	2.90

## Conclusion

In this study, two new crystal structures of 1,4-diazepine and its nitroso derivative(DIAZ1 & DIAZ2) were synthesized and characterized by X-ray crystallographic methods. Both 1,4-diazepine derivatives are crystallized in triclinic space group wherein the diazepine rings take up chair and boat conformations. In both the compounds, N-H…O hydrogen bonds lead to dimer formation. The molecules DIAZ1 and DIAZ2 are docked with the targeted protein NS5B RNA Polymerase and the results are compared with the cocrystallized ligand dibenzodiazepine.

## Additional files

Additional file 1:
**Structural information (CIF) for DIAZ1.**
Additional file 2:
**Structural information (CIF) for DIAZ2.**
